# Preclinical Metrics Correlate With Drug Activity in Phase II Trials of Targeted Therapies for Non-Small Cell Lung Cancer

**DOI:** 10.3389/fonc.2020.587377

**Published:** 2020-11-05

**Authors:** Brad Rybinski, H. Dean Hosgood, Sara L. Wiener, Daniel A. Weiser

**Affiliations:** ^1^ Department of Internal Medicine, University of Maryland Medical Center, Baltimore, MD, United States; ^2^ Department of Epidemiology and Population Health, Albert Einstein College of Medicine, Bronx, NY, United States; ^3^ Albert Einstein College of Medicine, Bronx, NY, United States; ^4^ Departments of Pediatrics & Genetics, Albert Einstein College of Medicine, Bronx, NY, United States; ^5^ Division of Pediatric Hematology, Oncology, and Marrow & Blood Cell Transplantation, Children’s Hospital at Montefiore, Bronx, NY, United States

**Keywords:** targeted therapy, mouse model, patient derived (tumor) xenograft model, co-clinical trials, non-small cell lung cancer

## Abstract

Novel oncology drugs often fail to progress from preclinical experiments to FDA approval. Therefore, determining which preclinical or clinical factors associate with drug activity could accelerate development of effective therapies. We investigated whether preclinical metrics and patient characteristics are associated with objective response rate (ORR) in phase II clinical trials of targeted therapies for non-small cell lung cancer (NSCLC). We developed a reproducible process to select a single phase II trial and supporting preclinical publication for a given drug-indication pair, which we defined as the pairing of a small molecule inhibitor (e.g., crizotinib) with the specific patient population for which it was designed to work (e.g., patients with an *ALK* aberration). We demonstrated that robust drug activity in mice, as measured by change in tumor size, is independently associated with improved ORR in phase II clinical trials. The number of mice utilized in experiments, the number of publications referencing the drug for NSCLC before the phase II clinical trial, and whether the drug was approved for a cancer other than NSCLC also significantly correlated with ORR. Among clinical characteristics, sex, race, histology, and smoking history were significantly associated with ORR. Further research into metrics that correlate with drug activity has the potential to optimize selection of novel therapies for clinical trials and enrich the drug development pipeline, particularly for patients with targetable genetic aberrations and rare cancers.

## Introduction

Despite the substantial success of some molecularly targeted therapies, most new oncology compounds fail to progress from preclinical studies to successful phase III trials (1), with one recent study estimating that only 3.4% of investigational agents achieve FDA approval (2). The high rate of drug failure also contributes significantly to the $2.6 billion cost of bringing a single novel therapeutic to market (3, 4). This low success rate has many causes, but one common explanation is that clinical trials are backed by inadequate preclinical research that relies on mouse models which do not sufficiently resemble human patients and disease (5–7). Yet, individualized patient derived xenograft (PDX) mouse models can match patient treatment response (8–10), and “co-clinical trials” have had modest success (11–13). These results clearly suggest that mouse models can predict oncology drug efficacy, but the low success rate of novel agents in clinical trials remains.

Inspired by this contradiction, we sought to determine whether the phase II activity of small molecule inhibitors for non-small cell lung cancer (NSCLC) was associated with preclinical metrics that reflect the quality of early preclinical literature. We chose NSCLC as our cancer of interest because NSCLC has a large number of driver mutations for which many molecularly targeted agents have been developed (14, 15). Therefore, NSCLC provides a robust sample of targeted agents with different preclinical and clinical efficacies. Preclinical metrics investigated for an association with phase II activity included features of mouse experiments, such as drug activity in mice as measured by T/C ratio (the mean volume of the tumors in mice who received treatment/the mean volume of the tumors in mice who received control), as well as information that would have been readily available at the time of the phase II trial, such as the number of pre-phase II publications investigating the drug in NSCLC. We then developed a reproducible methodology to select a single phase II trial and supporting preclinical publication for a given drug-indication pair (DIP), which we defined as the pairing of a small molecule inhibitor designed to inhibit a specific oncoprotein (e.g. EGFR with exon 19 deletion) or oncogenic process (e.g. angiogenesis) with the specific patient population for which it was designed to work. This methodology allowed us to then correlate preclinical variables and the objective response rate (ORR) observed in the phase II trial. Any association could offer new insight into prioritization of drugs for clinical development.

We also investigated phase II study patient clinical characteristics, such as smoking history and prior chemotherapy exposure, and determined whether they associated with ORR within our sample of phase II trials. We selected these factors not only because they are well described in the majority of NSCLC clinical trials, but also because demographic factors such as race (16), sex (17), performance status (18, 19), histology (18), and smoking history (16) have been associated with differences in NSCLC survival. The mechanisms by which these characteristics affect survival are undoubtedly complex and multifactorial, but any association between phase II activity and a demographic characteristic would implicate different biological responses to molecularly targeted agents as one cause of heterogeneous outcomes. Such a relationship between demographic factors and ORR would suggest that preclinical work could better predict therapeutic efficacy if designed to better reflect the diverse patient populations of clinical trials.

In this study, we demonstrated that activity in mice, as measured by T/C ratio, is independently associated with ORR in phase II trials. Additional preclinical metrics and clinical characteristics also associate with ORR. Our findings highlight the value of rigorous preclinical investigation and suggest that successful therapeutics are backed by robust preclinical research.

## Materials and Methods

### Selection and Definition of Clinical Characteristics and Preclinical Metrics

We selected 17 clinical characteristics to extract from phase II studies and 8 preclinical metrics to extract from preclinical publications and literature research. The clinical characteristics consisted of those features of the patient population commonly listed in “Table 1” of a phase II study investigating a novel lung cancer therapy (sex, race, performance status, smoking history, etc.). Preclinical metrics consisted of data from mouse experiments: the T/C ratio (the mean volume of the tumors in mice who received treatment/the mean volume of the tumors in mice who received control, at the end of the study), whether the T/C ratio is equal to 0, the duration of time that treated mice were monitored for tumor size, and the number of mice treated with drug. T/C ratio was selected as a measure of drug activity in mice, and whether the T/C ratio was equal to zero was included as a marker of dramatic activity (i.e. if the T/C ratio is zero then complete tumor regression in mice was observed) (20). The duration of time that treated mice were monitored for tumor size was selected to ask whether longer periods of time, which would presumably allow for a greater chance of tumor recurrence or re-growth, correlated with drug activity or FDA approval. The number of mice treated with drug was selected to assess if the treated mouse sample size correlated with activity or approval, as a sufficiently large sample size may be necessary to optimally reflect variable human activity. We also noted whether the drug was approved for cancer types other than NSCLC at the time of the selected phase II trial (to ask if there had been historical utility in re-purposing other cancer drugs for NSCLC), the type of mouse model (human cell line xenograft, patient derived xenograft, genetically engineered mouse model, or mouse cell line xenograft, to attempt to ask whether one type of mouse model better reflected activity in humans), and the type of patient and mouse model matching. For the type of patient and mouse model matching, cases in which the mouse model precisely matched the patients in terms of molecular subtype were classified as “selected to selected”, cases in which a new targeted therapy was tested in patients and mice resistant to another targeted therapy (e.g. ceritinib for crizotinib resistant patients) were classified as “resistant to resistant”, and cases in which neither patients nor mice were selected by their molecular subtype were classified as “unselected to unselected”. This metric was included to evaluate whether the similarity between mouse and patient tumors with respect to molecular matching correlated with ORR or drug approval. Presumably, “selected to selected” DIPs demonstrated molecular matching as similarly as possible, “resistant to resistant” DIPs demonstrated molecular matching that was potentially less similar (as resistance could be generated by a known mutation or empirically), and “unselected to unselected” DIPs demonstrated the least similar matching, as agents such as angiogenesis inhibitors are not given based on molecular characterization in either patients or mice. Finally, to measure early interest in a drug from the research community, we used PubMed to search “(drug name) AND lung cancer” (e.g. (gefitinib) AND lung cancer) and recorded the number of publications that were present up to the date that the selected phase II trial was published. We incorporated this metric to assess whether experts’ belief in the potential of a novel therapy, as reflected by more researchers studying and writing about the drug, has a relationship to the actual activity of the drug when it is eventually tested in human trials.

### Selection of Approved and Unapproved Drug-Indication Pairs

A drug-indication pair was defined as the match between a small molecule inhibitor designed to inhibit a specific target (e.g. mutant EGFR with exon 19 deletion) or oncogenic process (e.g. angiogenesis) with the patient population for which it was designed to work. For most drugs, the patient population was molecularly defined by a mutation within an oncogene. The same drug could produce more than one potential drug-indication pair; for example, gefitinib produced a potential drug-indication pair for patients with *EGFR* exon 19 deletion and patients with *EGFR* L858R mutation. For some drugs, such as angiogenesis inhibitors, a molecular patient population was not defined, and in that case the drug-indication pair consisted of the drug and “unselected patients”. FDA approved drug-indication pairs were identified by searching the National Library of Medicine *via* DailyMed (https://dailymed.nlm.nih.gov/dailymed/) for targeted small molecule inhibitors that were approved for NSCLC as of June 1^st^, 2019. Drugs granted accelerated approval were eligible for inclusion. Approved drug-indication pairs in which no preclinical study was available were excluded. For unapproved drugs, we searched PubMed for phase II studies of small molecule inhibitors tested in NSCLC between 1/1/06-12/31/15, with the rationale that any unapproved drugs tested during this time period are unlikely to gain approval in the near future. The same unapproved drug was often tested for more than one patient population or molecular indication. We therefore defined the indication for unapproved drugs as that indication which 1) produced the highest ORR 2) produced acceptable toxicity as judged by the study authors and 3) had an available preclinical publication.

### Selection of Phase II Clinical Trials and Publications Describing Preclinical Mouse Experiments

When multiple phase II trials for a given drug-indication pair were available, the trial with the greatest number of patients who received the investigational drug was selected. When there were two studies in which the difference in the number of patients who received the drug was less than 10%, studies conducted in the United States or globally were selected over studies conducted exclusively in Asia in order to maximize patient ethnic diversity. To identify preclinical publications with mouse experiments, we searched PubMed using the search term “(drug name) AND lung cancer” (e.g. (gefitinib) AND lung cancer) for preclinical studies that 1) appropriately matched the patient population and indication of the clinical trial 2) utilized mouse models and 3) were published before the phase II study or within one year after the date of its publication. An appropriate date filter was used to identify studies that met criterion 3, and assessment for criteria 1 and 2 was performed by manual review. We required that preclinical studies be published before or within one year after publication of the phase II study in order to enrich for early, yet high-quality, publications.

An appropriate preclinical study used a mouse model that precisely matched the molecular subtype of the patients in the phase II trial (i.e., tumors in patients and mice both harbored *EGFR* mutant L858R), gave the mouse the drug being investigated in addition to any combination treatment that the patient received (if the patients received an investigational drug in combination with chemotherapy the mice did as well), and matched status of known resistance to targeted therapy. If the patients in the phase II study had received chemotherapy prior to receiving the investigational drug, the mice did not also have to receive the same prior chemotherapy. However, if the new drug was administered to the phase II patients in combination with chemotherapy as part of the study protocol, the mice were required to also receive the same chemotherapy drug. Resistance in mice could be generated by a specific mutation known to confer resistance or empirically, just as patients in the phase II study of an investigational agent designed to overcome resistance could have known resistance conferring mutations or empiric resistance evidenced by progression on prior treatment. In the rare instance in which multiple preclinical studies met criteria, the study with the most citations on Google Scholar was selected in order to enrich for high quality publications.

### Data Collection From Phase II Clinical Trials and Preclinical Publications

We collected data on drug activity and clinical characteristics from phase II clinical trials. Data on drug activity consisted of ORR (equal to the sum of complete and partial responses) and the percent of patients obtaining stable disease (% SD) in the appropriate patient population as defined by the drug-indication pair. Some clinical trials reported activity data at more than one dose of drug. In these instances, we recorded the highest ORR that resulted when a drug was given at a dose that produced acceptable toxicity as determined by the phase II study authors. ORR assessed by independent review was selected over ORR assessed by investigator review. Data on stable disease was often not able to be obtained, as study authors often reported only the ORR in patients with specific mutations. With the exception of N-selected patient population, which is the size of the patient population we extracted activity data from, data for all clinical characteristics was obtained from the entire patient population in the study, no matter what treatment they received. A positive smoking history was defined as the sum of current and former smokers, and it was calculated by summing these values or by subtracting the percent of never smokers from 100%. Data for all other clinical characteristics was able to be extracted directly from the publication, usually from Table 1. A clinical characteristic, such as the presence of brain metastasis, was recorded as zero if it was listed in a trial’s exclusion criteria.

All data from pre-clinical publications was obtained from experiments that utilized mouse models. When multiple appropriate mouse models were available within the preclinical study, the mouse model with the best (smallest) T/C ratio was selected and data was collected from this experiment.

Definitions and methods for collecting all of the pre-clinical metrics utilized in our study are described below.

T/C Ratio (%): T/C Ratio represents mean mouse tumor size in the treatment group divided by the control group, at the timepoint representing the end of the study. Smaller T/C ratios indicate a larger treatment response. Depending on data reporting in the individual studies, T/C Ratio was calculated by one of the following formulas:

Formula 1: T/C = (mean volume of the tumors in mice who received treatment at the end of the study/mean volume of the tumors in mice who received control at the end of the study) x 100%

Mean tumor sizes were typically extracted from graphical representations.

Formula 2: T/C = 1- % tumor growth inhibition

Tumor volume was typically calculated in the selected studies by caliper measurements, but assessment by radiological imaging was also acceptable. Activity reported as a “complete response”, “cure”, or similar was recorded as a T/C ratio of zero.

T/C Ratio = 0: Binary variable; reflects whether T/C = 0 or T/C >0.

Duration Animal Monitoring (days): The length of time that tumor size was monitored in the selected mouse experiment. Typically, this was the time point (X axis value) at which the T/C ratio was calculated.

N Mice: In the selected mouse experiment, the number of mice who received treatment. When the number of mice was described as a range (i.e. 6-8), the lower end of the range was recorded as the number of mice (i.e. 6 mice).

N Pubs Before Date of Phase II: The number of publications indexed on PubMed investigating the use of the drug in lung cancer as of the date that the phase II clinical trial was published. We searched “(drug name) AND lung cancer” (e.g. (gefitinib) AND lung cancer) in PubMed, using an appropriate date filter, and recorded the number of search results.

Mouse Model: The type of mouse model as defined by the authors of the study. There were four possibilities: human cell line xenograft, patient derived xenograft (PDX), genetically engineered mouse model (GEM), or mouse cell line xenograft.

Drug Is Approved for Other Cancers Before Phase II: We identified through internet research whether the drug was approved for use in other cancer types on the date that the phase II trial we identified was published.

Matching Type: Cases in which the mouse model precisely matched the patients in terms of molecular subtype were classified as “selected to selected” (e.g., patient tumors have *EGFR* exon 19 deletion, mouse tumor has *EGFR* exon 19 deletion), cases in which a new targeted therapy was tested in patients and mice resistant to another targeted therapy (e.g. ceritinib for crizotinib resistant patients and crizotinib resistant mice) were classified as “resistant to resistant”, and cases in which neither patients nor mice were selected by their molecular subtype or resistance status were classified as “unselected to unselected”.

Independent Replication: We utilized the random number generator available on Google to select five approved drug-indication pairs and five unapproved drug-indication pairs for independent replication. An independent reviewer (SW) then attempted to identify all publications and collect all data for these 10 drug-indication pairs. Her results were compared to the data collected and reported herein to assess for a statistically significant difference.

### Statistical Analysis

Descriptive statistics were produced for all clinical variables and pre-clinical metrics in each cohort of drug-indication pairs. T-tests were utilized to compare the means of continuous variables, and Chi-squared tests were used to compare counts among categorical variables. We compared FDA approved drug-indication pairs to FDA unapproved drug-indication pairs, and we compared drugs with a high ORR to drugs with a low ORR (without consideration to approval status). Drugs defined as having a high ORR had an ORR of ≥ 50%, with the rationale that a drug which produces responses in half of patients or more can be considered to demonstrate robust clinical activity. For univariate analysis, we calculated the coefficient of determination (R^2^) and the corresponding p value. For multivariate analysis, we performed multivariate linear regression analysis for continuous dependent variables and binary logistic regression for categorical dependent variables. For all tests, a p value of <0.05 was considered significant. Outliers were identified by visual inspection of scatter plots. When outliers were considered present, analysis was performed with and without them. All statistical analysis was performed on SAS version 9.4 or GraphPad Prism version 7.04.

## Results

### A Reproducible Process for Identifying Phase II Clinical Trials and Supporting Preclinical Publications

We identified 44 drug-indication pairs (DIPs), of which 29 met criteria for inclusion in the study cohort (**Table S1**). Excluded DIPs consisted of nine unapproved DIPs and six approved DIPs (**Table S2**). All excluded DIPs lacked a mouse model that sufficiently matched the patient population studied in the selected phase II trial. The study cohort was nearly matched with respect to FDA approval; FDA approved DIPs (“Approved DIPs”) accounted for 14 of the included DIPs, and non-FDA approved DIPs (“Unapproved DIPs”) accounted for 15 of the included DIPs. Both approved and unapproved DIPs included frontline targeted therapies as well as second line therapies designed to overcome resistance to prior treatment with targeted agents (i.e. osimertinib and rociletinib for disease harboring *EGFR* T790M mutation). Only unapproved DIPs contained instances in which unselected patients were matched to unselected mice. Cases of unselected matching included 5 angiogenesis inhibitors and the polo-like kinase 1 inhibitor BI-2536.

All publications from which data was collected are listed in **Table S1**. After all data had been extracted, a collaborator who was not involved in the data collection process replicated the process of selecting studies and collecting data for 10 randomly selected DIPs. There was no statistically significant difference between the data from the study cohort and the data collected during the replication attempt (**Table S3**).

### Clinical Characteristics of the Phase II Patient Population Associate With Drug Activity

To determine whether clinical characteristics associate with drug activity, we compared the patient characteristics reported in studies demonstrating a high ORR (ORR ≥50%, ORR High) to the patient characteristics reported in studies demonstrating a lower ORR (ORR <50%, ORR Low). We also compared the patient characteristics reported in studies of FDA approved DIPs (“Approved”) to those reported in studies of unapproved DIPs (“Unapproved”). As expected, Approved DIPs reported a higher ORR than Unapproved DIPs (**Table 1**), and groups with a significantly higher ORR (Approved DIPs and ORR ≥ 50%) reported a significantly lower rate of stable disease (**Tables 1** and **2**). Approved DIPs were tested in significantly larger patient populations than Unapproved DIPs in terms of the total number of patients enrolled in the study (**Table 1**, termed “N-intention to treat”) and the number of patients who received the drug for the indication specified in our analysis at the dose that produced the highest ORR (**Table 1**, termed “N-selected patient population”). Approved DIPs and ORR High DIPs both had significantly more Asian patients and cases of adenocarcinoma than Unapproved DIPs and ORR Low DIPs (**Tables 1** and **2**, respectively). Approved DIPs and ORR High DIPs also had fewer white patients, cases of squamous carcinoma, and patients with a positive smoking history (**Tables 1** and **2**, respectively).

**Table 1:** Patient characteristics differ significantly between Phase II studies of FDA Approved and Unapproved drug-indication pairs.

Approved vs. Unapproved	Unapproved Mean (sd)	Approved Mean (sd)	p value
% ORR	26.95 (25.43)	65.14 (17.72)	**<.0001**
N-intention to treat*	91.00 (48.02)	154.5 (90.67)	**0.0246**
N-selected patient population**	38.26 (26.97)	89.1429 (58.22)	**0.0050**
% Stable Disease	43.01 (19.68)	23.32 (9.83)	**0.0220**
% Female	53.97 (13.99)	61.69 (8.67)	0.0969
Median age	62.62 (2.97)	58.19 (5.90)	**0.0237**
% White	82.87 (10.19)	46.09 (17.94)	**<.0001**
% Asian	19.80 (29.38)	44.74 (21.89)	**0.0336**
% ECOG = 0	43.28 (19.27)	38.11 (6.39)	0.4517
% ECOG = 1	54.64 (19.52)	55.41 (6.18)	0.9109
% ECOG = 2	1.50 (3.41)	6.14 (5.31)	**0.0348**
% Patients who are current or former smokers	77.22 (18.87)	32.28 (8.42)	**<.0001**
% Adenocarcinoma	76.07 (17.59)	96.21 (3.31)	**0.0019**
% Squamous carcinoma	12.21 (8.64)	0.87 (0.72)	**0.0037**
% Patients with CNS metastasis	22.00 (25.40)	38.95 (21.43)	0.2031
% Patients who received prior chemotherapy	54.92 (48.41)	60.26 (43.24)	0.7732
% Stage IV	79.70 (29.32)	87.90 (13.50)	0.5675
% Chemo less than 5%	41.67%	30.77%	0.5706

P values in bold are significant.

*”N-intention to treat” refers to the total number of patients enrolled in the trial, no matter whether they received the investigational drug or not. **”N-selected patient population” refers to the number of patients who received the drug for the indication specified in our analysis at the dose that produced the highest objective response rate (ORR).

ORR High DIPs had significantly more female patients (**Table 2**) and there was a similar trend toward significance with respect to Approved DIPs (**Table 1**). Median age and the percentage of patients with a performance status of ECOG 2 differed significantly between Approved DIPs and Unapproved DIPs, but the differences were quite small in magnitude (**Table 1**). There were no significant differences with respect to the frequency of prior chemotherapy, stage IV disease, or CNS metastasis (**Tables 1** and **2**). ORR was positively correlated with female sex (p=0.0002), Asian ethnicity (p< 0.0001), and adenocarcinoma (p=0.0010), and negatively correlated with White ethnicity (p< 0.0001), squamous histology (p=0.0062), and smoking status (p< 0.0001) (**Figure S1**).

**Table 2:** Patient characteristics differ significantly between Phase II studies of objective response rate (ORR) High (ORR ≥ 50%) and ORR Low (ORR <50%) drug-indication pairs.

ORR ≥ 50% v. ORR < 50%	ORR < 50%(ORR Low)Mean (sd)	ORR ≥ 50%(ORR High)Mean (sd)	p value
N-intention to treat*	107.9 (68.65)	132.8 (84.61)	0.3998
N-selected patient population**	61.23 (42.06)	64.13 (58.71)	0.8826
% Stable Disease	43.80 (18.41)	22.43 (10.50)	**0.0113**
% Female	48.92 (8.99)	65.03 (9.57)	**0.0001**
Median age	61.73 (5.26)	59.43 (4.93)	0.2655
% White	77.70 (15.87)	47.58 (20.93)	**0.0019**
% Asian	15.88 (14.90)	45.54 (28.84)	**0.0106**
% ECOG = 0	46.50 (19.23)	36.12 (8.12)	0.1173
% ECOG = 1	50.47 (17.94)	59.06 (11.03)	0.1972
% ECOG = 2	2.48 (4.63)	4.80 (5.12)	0.3160
% Patients who are current or former smokers	82.88 (8.86)	32.25 (7.95)	**<.0001**
% Adenocarcinoma	74.26 (16.97)	96.35 (3.69)	**0.0004**
% Squamous carcinoma	11.71 (9.11)	1.59 (2.48)	**0.0123**
% Patients with CNS metastasis	32.85 (38.22)	35.62 (18.08)	0.8399
% Patients who received prior chemotherapy	55.46 (46.39)	60.12 (45.16)	0.8018
% Stage IV	78.22 (30.74)	88.75 (12.09)	0.4432
% Chemo less than 5%	38.46%	33.33%	0.7896

P values in bold are significant.

*”N-intention to treat” refers to the total number of patients enrolled in the trial, no matter whether they received the investigational drug or not. **”N-selected patient population” refers to the number of patients who received the drug for the indication specified in our analysis at the dose that produced the highest ORR.

### Preclinical Metrics Associate With Drug Activity in Phase II Clinical Trials

**Figure 1:**
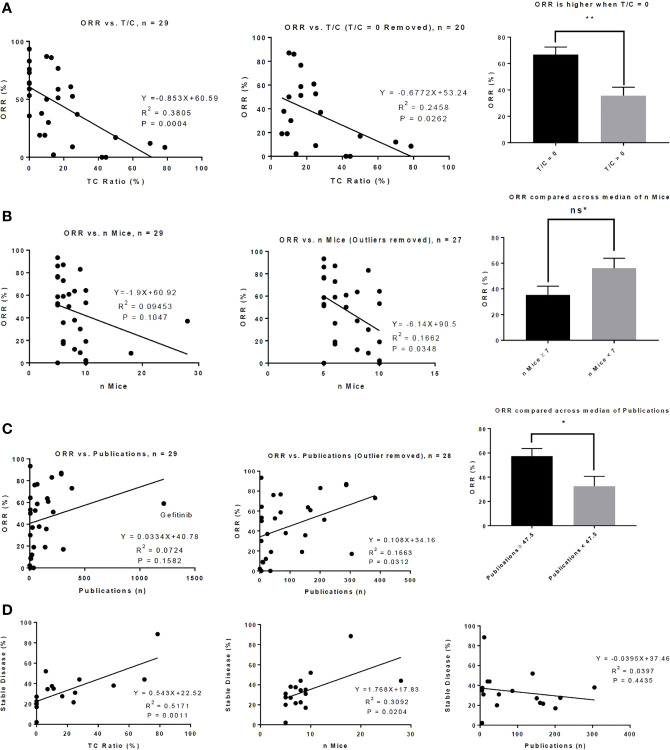
Preclinical metrics associate with activity in phase II clinical trials. **(A)** There is a significant correlation between T/C Ratio and objective response rate (ORR) (left) and this correlation remains when drugs that demonstrated a T/C Ratio of 0 in mouse models were removed from the analysis (middle). Drugs that produced a T/C ratio of 0 in mouse models produced a significantly higher ORR in phase II clinical trials (right). **(B)** There is a modest correlation between the number of mice who were treated with a drug and the ORR of the drug in phase II clinical trials (left); this correlation becomes significant when 2 outliers were removed (middle). The median number of mice treated with drug is 7. There was a trend toward significance in which drugs tested in 7 or more mice produced a lower ORR than drugs tested in fewer than 7 mice. **(C)** There is a correlation between ORR and the number of publications indexed on PubMed up to the date the phase II clinical trial was published (left); this correlation is increased when 1 outlier (gefitinib) was removed (middle). The median number of publications is 47.5; drugs in which 47.5 or more publications were indexed on PubMed up to the date of the phase II trial produced a significantly higher ORR. **(D)** Correlations between the percent of patients in the phase II trial who achieved stable disease and T/C ratio (left), number of mice who received drug (middle), and number of publications indexed on PubMed up to the date of the phase II trial (right) are shown. ns* = trend toward significance with p = 0.0534, *P < 0.05, **P < 0.01, Bar graphs represent means with standard error.

To investigate whether a variety of preclinical metrics associate with drug activity, we compared preclinical data across ORR High and ORR Low as well as Approved and Unapproved (**Table 3**). DIPs with greater activity in phase II clinical trials also demonstrated greater activity in mice. To quantify drug activity in mice, we utilized the T/C ratios reported in our set of preclinical publications. Smaller T/C ratios represent greater activity, with a T/C ratio of zero indicating complete tumor regression (see Methods). ORR High DIPs demonstrated a significantly smaller T/C ratio than ORR Low DIPs (8.24% vs. 29.63%, p=0.0043), and a significantly larger fraction of ORR High DIPs demonstrated a T/C ratio equal to zero (50% vs. 7.69%, p=0.0143). 8/9 DIPs that produced a T/C ratio of zero also produced an ORR ≥ 50%. There was a significant negative correlation between ORR and T/C ratio (R^2 ^= 0.3805, p=0.0004), which remained when drugs which demonstrated a T/C ratio of 0 were removed (R^2 ^= 0.2458, p=0.0262) (**Figure 1A**). Drugs with a T/C ratio of zero also demonstrated a significantly higher ORR (66.82% vs. 35.74%, p=0.0054, **Figure 1A**). T/C ratio was significantly smaller in Approved DIPs than Unapproved DIPs (9.21% vs. 25.87%, p=0.0303), though there was not a significant difference with respect to the fraction of drugs that demonstrated a T/C ratio of 0 (42.86% vs. 20.00%, p=0.1837).

**Table 3:** Preclinical metrics differ significantly between objective response rate (ORR) High and ORR Low drug-indication pairs as well as Approved and Unapproved drug-indication pairs.

ORR ≥ 50% v. ORR < 50%	ORR < 50(ORR Low)Mean (sd)	ORR ≥ 50(ORR High)Mean (sd)	p value
T/C Ratio	29.63 (25.49)	8.24 (9.35)	**0.0043**
Duration animal monitoring (days)	38.46 (37.27)	41.50 (36.13)	0.8259
N mice	10.23 (6.26)	6.50 (1.83)	**0.0310**
N pubs before date of Phase II	63.77 (88.43)	198.3 (296.4)	0.1267
T/C Ratio = 0	7.69%	50%	**0.0143**
Mouse model	HC = 76.93% PDX = 0% GEM = 7.69% MC = 15.38%	HC = 81.25% PDX = 12.5% GEM = 6.25% MC = 0%	0.2483
Drug is approved for other cancers before Phase II	30.77%	6.25%	0.0821
Matching type	Selected to selected = 38.47% Resistant to Resistant = 15.38% Unselected to unselected = 46.15%	Selected to selected = 87.5% Resistant to Resistant = 12.5% Unselected to unselected = 0%	**0.0065**
**Approved vs. Unapproved**	**Unapproved** **Mean (sd)**	**Approved** **Mean (sd)**	**p value**
			
T/C Ratio	25.87 (25.66)	9.21 (9.45)	**0.0303**
T/C Ratio = 0	20.00%	42.86%	0.1837
Mouse model	HC = 73.33% PDX = 6. 67% GEM = 6.67% MC = 13.33%	HC = 85.70% PDX = 7.15% GEM = 7.15% MC = 0%	0.5700
Duration animal monitoring (days)	36.73 (34.90)	43.79 (38.13)	0.6073
N mice	10.33 (5.73)	5.86 (1.10)	**0.0079**
N pubs before date of Phase II	54.07 (89.90)	227.9 (305.0)	**0.0439**
Drug is approved for other cancers before Phase II	33%	0	**0.0176**
Matching type	Selected to selected = 60% Resistant to Resistant = 0% Unselected to unselected = 40%	Selected to selected = 71.43% Resistant to resistant = 28.57% Unselected to unselected = 0%	**0.0066**

P values in bold are significant.

HC, human cell line xenograft; PDX, patient derived xenograft; GEM, genetically engineered mouse model; MC, mouse cell line xenograft.

Intriguingly, ORR High and Approved studies treated fewer mice than in ORR Low and Unapproved studies (**Table 3**). Though the correlation between the number of mice treated and ORR was small and did not achieve significance, there was a significant negative correlation when two outliers were removed (**Figure 1B**). When the number of mice was stratified by its median (seven mice) there was nearly a significant difference in ORR above and below the median (≥7 mice vs. < 7 mice = 35.38% vs. 56.11%, p=0.0534, **Figure 1B**). There was no significant correlation between the number of mice treated and T/C ratio (R^2 ^= 0.0003, p=0.9279).

A larger early publication record was suggestive of drug success. Approved DIPs had a significantly higher number of publications published before the date on which the phase II trial was published (227.9 vs. 54.07, p=0.0439), and there was a trend toward significance in which ORR High DIPs had more publications than ORR Low DIPs (198.3 vs. 63.77, p=0.1267) (**Table 3**). Although initially there was no significant correlation between ORR and the number of early publications, when one outlier (gefitinib) was removed there was a significant positive correlation between ORR and publication record (**Figure 1C**). ORR was also significantly higher when the number of publications was greater than the median (57.32% vs. 32.48%, p=0.0228, **Figure 1C**). There was a significant difference in the distribution of matching type between ORR High and ORR Low and Approved vs. Unapproved (**Table 3**), with unselected matches isolated to the ORR Low and Unapproved groups. Among those DIPs for which data on the percent of patients achieving stable disease was available, there was a significant association between stable disease and T/C ratio, as well as stable disease and the number of treated mice (**Figure 1D**). There was no significant correlation between stable disease and the number of early publications (**Figure 1D**).

Approval for other cancer types was more common in drugs that were less successful for treatment of NSCLC. A significantly greater fraction of Unapproved DIPs contained drugs that were approved for treatment of other types of cancer before the phase II trial investigating their use in NSCLC (**Table 3**). More ORR Low drugs were also approved for other types of cancer than ORR High Drugs; the difference trended toward significance (**Table 3**).There was no difference between ORR High and ORR Low or Approved and Unapproved with respect to the duration of mouse monitoring or the frequency at which different types of mouse model were utilized (**Table 3**). However, 23/29 DIPs in our study reported results from human cell line xenograft models, with the remaining DIPs utilizing genetically engineered mouse models (n=2), PDX models (n=2), and mouse cell line xenograft models (n=2).

We reasoned that our inclusion of DIPs which matched unselected mice (mouse tumors not characterized with respect to mutation) and unselected patients (patient tumors not characterized with respect to mutation) could have affected the relationship we observed between preclinical metrics and drug activity. To this end, we excluded the 6 drugs (5 angiogenesis inhibitors and 1 polo-like kinase inhibitor) that matched unselected mice to unselected patients from our cohort and repeated our comparison of preclinical data across ORR High and ORR Low as well as Approved and Unapproved. We then compared the results of this analysis (termed Matched Drugs, n=23) to the results obtained from analysis of our entire cohort (termed All Drugs, n=29). When restricting to matched drugs, we observed similar results as seen in the larger cohort (**Tables S4** and **S5**). This finding suggests that the relationships between preclinical metrics and ORR or Approval Status remain in a strictly defined sample in which all patient tumors carry the same target mutation as the mouse models used to evaluate drug activity.

### T/C Ratio Is Independently Associated With ORR in Phase II Clinical Trials

Having established that certain preclinical metrics correlate with ORR in phase II clinical trials, we sought to establish which if any preclinical metrics are independently associated with ORR. Of the 8 preclinical metrics, we selected those with a p value of <0.20 when comparing ORR High to ORR Low to include in a multivariate linear regression model. We also excluded T/C ratio of zero, as it is collinear with T/C ratio. Multivariate analysis showed that only T/C ratio and Matching Type were independently associated with ORR (**Table 4**, ORR All Drugs). When DIPs that matched unselected mice to unselected patients were excluded from the model, only T/C ratio remained significant (**Table 4**, ORR Matched Drugs). We attempted to adjust our model for those clinical characteristics which were associated with ORR (sex, race, histology, and smoking history), as well as for prior chemotherapy exposure. In the adjusted analysis, no preclinical metrics were independently significant. However, due to lack of universal reporting of clinical trial data, only 11/29 DIPs contained data for all clinical characteristics and were able to be included in the adjusted analysis, suggesting that the adjusted analysis failed to capture the majority of our cohort. By logistic regression analysis, no preclinical variable was independently associated with drug approval status or an ORR below a threshold of 50% (**Tables S6** and **S7**).

**Table 4:** In multivariate analysis, only T/C ratio is independently associated with objective response rate (ORR) in matched and unmatched drug-indication pairs.

ORR All Drugs R^2^: 0.5625 Adjusted R^2^: 0.4674 F Value: 5.91 Pr > F: 0.0012		
**Predictor Variable**	**β Estimate (standard error)**	**p value**
T/C Ratio	−0.681 (0.285)	**0.0255**
N mice	−0.702 (0.916)	0.4510
N pubs before date of Phase II	0.007 (0.018)	0.6780
Drug is approved for other cancers before Phase II?	4.627 (14.345)	0.7500
Matching Type	−14.319 (5.107)	**0.0101**
**ORR Matched Drugs** **R^2^:** 0.5119 **Adjusted R^2^:** 0.3683 **F Value:** 3.57 **Pr > F: 0.0218**		
**Predictor Variable**	**β Estimate (standard error)**	**p value**
T/C Ratio	−0.902 (0.307)	**0.0092**
N mice	−1.232 (1.000)	0.2350
N pubs before date of Phase II	−0.002 (0.019)	0.9097
Drug is approved for other cancers before Phase II?	−5.404 (15.777)	0.7362
Matching Type	−16.948 (12.253)	0.1845

Matching type is independently significant for the All Drugs model. P values in bold are significant.

## Discussion

The FDA approval rate of promising novel cancer therapies is extremely low (1, 2). Late phase clinical trials that underperform subject patients to ineffective treatments and divert resources and patients from other promising studies. This can be particularly devastating for rare and underfunded malignancies (21), including many childhood cancers (22–24), for which clinical trial recruitment is already challenging. With a goal of precision and personalized medicine for patients, it is increasingly imperative to improve prediction modeling for which drugs are likely to succeed in clinical trials. We therefore attempted to identify preclinical factors that were associated with drug activity in phase II trials, focusing on novel agents tested in NSCLC.

We first developed a reproducible process to filter multiple clinical trials and preclinical publications investigating a novel therapy down to a seminal clinical trial with one supporting preclinical publication. We then correlated preclinical metrics and phase II ORR and found that drugs with greater activity in mice demonstrate greater activity in clinical trials. Most intriguingly, 8/9 DIPs that produced a T/C ratio of zero also produced an ORR ≥ 50%, suggesting that complete response to a drug in mice is highly suggestive of robust clinical activity. Furthermore, the mean ORR for drugs that produced a T/C ratio of zero was nearly twice as large as the ORR for drugs that did not (**Figure 1A**). We expect that the significant correlation we observed between T/C ratio and ORR results from molecular matching of mouse models to patients. For example, when comparing the ORR to targeted therapy in patients with *EGFR* exon 19 deletion to the T/C ratio of molecularly matched mouse tumors, there is a significant correlation between clinical and preclinical activity that dissipates for studies of conventional chemotherapy.

If there was no correlation between drug activity in mice and drug activity in clinical trials, there would be little purpose in utilizing mouse models. From this perspective, the correlation between T/C ratio in mice and ORR in humans that we observed is not surprising. However, the correlation is intriguing when the limitations of mouse models are considered. Differences in biology between mice and humans create potential for off-target effects that would not be apparent in mouse models; this could manifest as reduced activity or unanticipated toxicity when a drug tested in mice reaches the clinic (25). Mice routinely start treatment when they harbor a relatively low tumor burden and rarely harbor the metastatic disease that is often present in patients (7). Moreover, the microenvironment is suspected to differ substantially between mouse models and human patients (25), and the tumor microenvironment has emerged as powerful modulator of therapeutic activity (25, 26). Finally, chemotherapy regimens administered to patients are complex and may include multiple drugs and dose adjustments, in addition to combination with radiation and immunological therapies. These regimens are nearly impossible to replicate in mice (7).

To the best of our knowledge, little work has investigated whether T/C ratio correlates with ORR when targeted therapies are investigated in appropriately matched mouse models. Previously, Wong et al. tested 8 drugs, including both targeted therapies and conventional chemotherapeutics, in a heterogenous set of mouse models and observed no significant correlation between activity in mice and ORR in clinical trials when mice were given drugs at maximally tolerated doses (27). However, there was a significant correlation between human and mouse activity in mathematical simulations that adjusted for human drug exposures and pharmacokinetics (27). Additional work has been limited exclusively to chemotherapeutic agents. In 2003, Voskoglou-Nomikos et al. observed no significant correlation between T/C ratio and phase II ORR in studies of chemotherapy drugs that utilized human xenograft models (20). However, there was a significant correlation when the authors included only drugs for which more than one human xenograft model was available (20). Likewise, the National Cancer Institute observed that, among 39 chemotherapeutic drugs investigated, activity in 1/3 or more of xenograft models is modestly predictive of activity in clinical trials (28).

Our analysis also identified additional relationships between preclinical metrics and ORR. We were curious as to whether interest in a drug from the research community, assessed by publication volume, affected its probability of success in clinical trials. Our data suggest that there is a positive correlation between ORR and the number of PubMed results investigating the drug in NSCLC that were published up to the date of the phase II trial. We also observed an inverse relationship between the number of mice utilized in preclinical experiments and ORR. The reason for this is unclear, though it is unlikely that researchers increase the number of mice in their treatment group to help small effect sizes achieve statistical significance, as we observed no correlation between T/C ratio and the number of mice. No drugs that were approved for other cancers gained FDA approval for NSCLC. Although our study was not able to include some notable examples of drugs that are approved for NSCLC and other cancers, such as dabrafenib and trametinib, our results suggest that drug re-purposing should be viewed with caution unless there is the support of a plausible mechanism of action.

Within phase II trials, we observed that there was a significant correlation between ORR and patient clinical characteristics, including sex, race, histology, and smoking history. In large phase III trials comparing the efficacy of novel NSCLC therapies to chemotherapy, these factors have also been shown to modestly affect progression free survival (29–32), indicating that patient characteristics and heterogeneity affect the utility of targeted therapies. Our results further suggest that selected patient characteristics correlate directly with response to drug, likely through poorly described biological mechanisms. However, we were unable to adjust our preclinical multivariate models for clinical features, as few clinical trials reported comprehensive data for features of interest. The lack of universal reporting in clinical trials is a well-documented limitation of many studies (33, 34).We also suspect that if preclinical mouse models were more representative of the diverse spectrum of human disease, prediction of drug success in the clinic would improve. For instance, sex as a biologic variable is now required to be addressed in NIH-sponsored research (35).

### Limitations

Our work has important limitations. More effective drugs were not more likely to utilize certain types of mouse models (e.g., cell line xenograft, PDX), though 23/29 of the drug-indication pairs in our data set utilized human cell line xenograft models. By including only preclinical studies that had been published before or within 1 year of the selected Phase II trial, we focused on early literature that could in principle be used to prospectively predict clinical activity. However, this shifted our sample of preclinical studies toward older literature that utilized fewer PDX and GEM models. The relative ability of PDX, cell line xenograft, and GEM models to predict clinical activity is an intriguing question that warrants further investigation. We did not include monoclonal antibodies or immunotherapies, and metrics which correlate with activity among small molecule inhibitors may or may not correlate with the activity of these treatments. Among preclinical metrics, we did not incorporate *in vitro* measures of drug activity such as half maximal inhibitory concentration (IC_50_), and we did not consider measures of drug toxicity. These characteristics of a novel therapeutic agent play an important role in whether meaningful therapeutic efficacy and FDA approval are ultimately achieved. Our research was also focused on a single cancer type and the conclusions may not be generalizable to other cancers and therapies. Finally, our study was descriptive in nature and prospective validation of our findings is necessary.

### Conclusions

We have identified several metrics, particularly drug activity in mice as measured by T/C ratio, that correlate with drug activity in phase II clinical trials. Our results support rigorous preclinical investigation of promising anti-cancer therapies and suggest that preclinical research can inform the likelihood of drug success in the clinic. Future work may focus on determining whether our findings are generalizable across other cancer types. Our method could also be utilized to determine which preclinical metrics and clinical characteristics correlate with the activity of monoclonal antibodies and immunotherapies. We observed a significant correlation between drug activity and patient clinical characteristics, including sex, race, histology, and smoking history. Whether such clinical characteristics affect drug activity in other cancers is another intriguing prospect for further investigation. Predictive metrics for drug success, whether consisting of preclinical metrics or patient characteristics, could streamline drug development and guide prioritization for clinical use. Ultimately, these efforts have the potential to accelerate progress toward more effective treatments and improve patient outcomes.

## Data Availability Statement

The original contributions presented in the study are included in the article/**Supplementary Materials**. Further inquiries can be directed to the corresponding author.

## Author Contributions

BR played a role in data collection, data analysis, manuscript writing, manuscript editing, and conceptualization. HH played a role in data analysis, manuscript editing, and conceptualization. SW played a role in data collection and data analysis. DW played a role in manuscript writing, manuscript editing, and conceptualization. All authors contributed to the article and approved the submitted version.

## Funding

This work was supported by the Louis Ritter Memorial Fellowship to BR.

## Conflict of Interest

The authors declare that the research was conducted in the absence of any commercial or financial relationships that could be construed as a potential conflict of interest.
