# Supplementary material for: Preclinical Metrics Correlate With Drug Activity in Phase II Trials of Targeted Therapies for Non-Small Cell Lung Cancer
Source: Front Oncol. 2020 Nov 5;10:587377. doi: 10.3389/fonc.2020.587377 (PMC7674799; doi:10.3389/fonc.2020.587377)
Supplement: Supplementary file 1 [file Table_1.docx]

**Supplemental Table 1:** Drug-indication pairs (DIPs) included in the study, and the corresponding studies from which data was extracted.

|  | **Drug-Indication Pair** | **Phase II Clinical Trial** | **Pre-clinical Study** |
| --- | --- | --- | --- |
| **Approved** | Afatinib-*EGFR* exon 19 deletion | Park K, Tan EH, O'Byrne K, et al. Afatinib versus gefitinib as first-line treatment of patients with EGFR mutation-positive non-small-cell lung cancer (LUX-Lung 7): a phase 2B, open-label, randomised controlled trial. *Lancet Oncol.* 2016;17(5):577-589. | Stewart EL, Mascaux C, Pham NA, et al. Clinical Utility of Patient-Derived Xenografts to Determine Biomarkers of Prognosis and Map Resistance Pathways in EGFR-Mutant Lung Adenocarcinoma. *J Clin Oncol.* 2015;33(22):2472-2480. |
|  | Brigatinib-*ALK*+, crizotinib resistance | Kim DW, Tiseo M, Ahn MJ, et al. Brigatinib in Patients With Crizotinib-Refractory Anaplastic Lymphoma Kinase-Positive Non-Small-Cell Lung Cancer: A Randomized, Multicenter Phase II Trial. *J Clin Oncol.* 2017;35(22):2490-2498. | Katayama R, Khan TM, Benes C, et al. Therapeutic strategies to overcome crizotinib resistance in non-small cell lung cancers harboring the fusion oncogene EML4-ALK. *Proc Natl Acad Sci U S A.* 2011;108(18):7535-7540. |
|  | Osimertinib-*EGFR* T790M and exon 19 deletion | Goss G, Tsai CM, Shepherd FA, et al. Osimertinib for pretreated EGFR Thr790Met-positive advanced non-small-cell lung cancer (AURA2): a multicentre, open-label, single-arm, phase 2 study. *Lancet Oncol.* 2016;17(12):1643-1652. | Cross DA, Ashton SE, Ghiorghiu S, et al. AZD9291, an irreversible EGFR TKI, overcomes T790M-mediated resistance to EGFR inhibitors in lung cancer. *Cancer Discov.* 2014;4(9):1046-1061. |
|  | Osimertinib-*EGFR* T790M and L858R exon 21 mutation | Goss G, Tsai CM, Shepherd FA, et al. Osimertinib for pretreated EGFR Thr790Met-positive advanced non-small-cell lung cancer (AURA2): a multicentre, open-label, single-arm, phase 2 study. *Lancet Oncol.* 2016;17(12):1643-1652. | Cross DA, Ashton SE, Ghiorghiu S, et al. AZD9291, an irreversible EGFR TKI, overcomes T790M-mediated resistance to EGFR inhibitors in lung cancer. *Cancer Discov.* 2014;4(9):1046-1061. |
|  | Osimertinib-*EGFR* exon 19 deletion w/o T790M mutation | Ramalingam SS, Yang JC, Lee CK, et al. Osimertinib As First-Line Treatment of EGFR Mutation-Positive Advanced Non-Small-Cell Lung Cancer. *J Clin Oncol.* 2018;36(9):841-849. | Cross DA, Ashton SE, Ghiorghiu S, et al. AZD9291, an irreversible EGFR TKI, overcomes T790M-mediated resistance to EGFR inhibitors in lung cancer. *Cancer Discov.* 2014;4(9):1046-1061. |
|  | Osimertinib-*EGFR* L858R exon 21 mutation w/o T790M | Ramalingam SS, Yang JC, Lee CK, et al. Osimertinib As First-Line Treatment of EGFR Mutation-Positive Advanced Non-Small-Cell Lung Cancer. *J Clin Oncol.* 2018;36(9):841-849. | Cross DA, Ashton SE, Ghiorghiu S, et al. AZD9291, an irreversible EGFR TKI, overcomes T790M-mediated resistance to EGFR inhibitors in lung cancer. *Cancer Discov.* 2014;4(9):1046-1061. |
|  | Alectinib-*ALK*+, crizotinib Resistance | Yang JC, Ou SI, De Petris L, et al. Pooled Systemic Efficacy and Safety Data from the Pivotal Phase II Studies (NP28673 and NP28761) of Alectinib in ALK-positive Non-Small Cell Lung Cancer. *J Thorac Oncol.* 2017;12(10):1552-1560. | Sakamoto H, Tsukaguchi T, Hiroshima S, et al. CH5424802, a selective ALK inhibitor capable of blocking the resistant gatekeeper mutant. *Cancer Cell.* 2011;19(5):679-690. |
|  | Alectinib-*ALK*+, No crizotinib resistance (first line) | Seto T, Kiura K, Nishio M, et al. CH5424802 (RO5424802) for patients with ALK-rearranged advanced non-small-cell lung cancer (AF-001JP study): a single-arm, open-label, phase 1-2 study. *Lancet Oncol.* 2013;14(7):590-598. | Sakamoto H, Tsukaguchi T, Hiroshima S, et al. CH5424802, a selective ALK inhibitor capable of blocking the resistant gatekeeper mutant. *Cancer Cell.* 2011;19(5):679-690. |
|  | Ceritinib- *ALK*+, crizotinib Resistance | Crino L, Ahn MJ, De Marinis F, et al. Multicenter Phase II Study of Whole-Body and Intracranial Activity With Ceritinib in Patients With ALK-Rearranged Non-Small-Cell Lung Cancer Previously Treated With Chemotherapy and Crizotinib: Results From ASCEND-2. *J Clin Oncol.* 2016;34(24):2866-2873. | Friboulet L, Li N, Katayama R, et al. The ALK inhibitor ceritinib overcomes crizotinib resistance in non-small cell lung cancer. *Cancer Discov.* 2014;4(6):662-673. |
|  | Ceritinib- *ALK*+, No crizotinib resistance (first line) | Felip E, Orlov S, Park K, et al. Phase 2 study of ceritinib in ALKi-naïve patients (pts) with ALK-rearranged (ALK+) non-small cell lung cancer (NSCLC): Whole body responses in the overall pt group and in pts with baseline brain metastases (BM). *Annals of Oncology.* 2016;27(suppl_6). | Friboulet L, Li N, Katayama R, et al. The ALK inhibitor ceritinib overcomes crizotinib resistance in non-small cell lung cancer. *Cancer Discov.* 2014;4(6):662-673. |
|  | Dacomitinib- *EGFR* exon 19 deletion | Janne PA, Ou SI, Kim DW, et al. Dacomitinib as first-line treatment in patients with clinically or molecularly selected advanced non-small-cell lung cancer: a multicentre, open-label, phase 2 trial. *Lancet Oncol.* 2014;15(13):1433-1441. | Engelman JA, Zejnullahu K, Gale CM, et al. PF00299804, an irreversible pan-ERBB inhibitor, is effective in lung cancer models with EGFR and ERBB2 mutations that are resistant to gefitinib. *Cancer Res.* 2007;67(24):11924-11932. |
|  | Crizotinib-*ALK*+ | Camidge DR, Bang YJ, Kwak EL, et al. Activity and safety of crizotinib in patients with ALK-positive non-small-cell lung cancer: updated results from a phase 1 study. *Lancet Oncol.* 2012;13(10):1011-1019. | Sang J, Acquaviva J, Friedland JC, et al. Targeted inhibition of the molecular chaperone Hsp90 overcomes ALK inhibitor resistance in non-small cell lung cancer. *Cancer Discov.* 2013;3(4):430-443. |
|  | Gefitinib- *EGFR* exon 19 deletion | Sequist LV, Martins RG, Spigel D, et al. First-line gefitinib in patients with advanced non-small-cell lung cancer harboring somatic EGFR mutations. *J Clin Oncol.* 2008;26(15):2442-2449. | Naumov GN, Nilsson MB, Cascone T, et al. Combined vascular endothelial growth factor receptor and epidermal growth factor receptor (EGFR) blockade inhibits tumor growth in xenograft models of EGFR inhibitor resistance. *Clin Cancer Res.* 2009;15(10):3484-3494. |
|  | Lorlatinib-*ALK*+, Resistance to crizotinib + an additional *ALK* inhibitor, resistance to alectinib, or resistance to ceritinib | Solomon BJ, Besse B, Bauer TM, et al. Lorlatinib in patients with ALK-positive non-small-cell lung cancer: results from a global phase 2 study. *Lancet Oncol.* 2018;19(12):1654-1667. | Zou HY, Friboulet L, Kodack DP, et al. PF-06463922, an ALK/ROS1 Inhibitor, Overcomes Resistance to First and Second Generation ALK Inhibitors in Preclinical Models. *Cancer Cell.* 2015;28(1):70-81. |
|  |  |  |  |
| **Unapprove**d | Rociletinib- *EGFR* T790M and exon 19 deletion | Sequist LV, Soria JC, Goldman JW, et al. Rociletinib in EGFR-mutated non-small-cell lung cancer. *N Engl J Med.* 2015;372(18):1700-1709. | Walter AO, Sjin RT, Haringsma HJ, et al. Discovery of a mutant-selective covalent inhibitor of EGFR that overcomes T790M-mediated resistance in NSCLC. *Cancer Discov.* 2013;3(12):1404-1415. |
|  | Rociletinib- *EGFR* T790M and L858R exon 21 mutation | Sequist LV, Soria JC, Goldman JW, et al. Rociletinib in EGFR-mutated non-small-cell lung cancer. *N Engl J Med.* 2015;372(18):1700-1709. | Walter AO, Sjin RT, Haringsma HJ, et al. Discovery of a mutant-selective covalent inhibitor of EGFR that overcomes T790M-mediated resistance in NSCLC. *Cancer Discov.* 2013;3(12):1404-1415. |
|  | Sorafenib in combination/erlotinib- *EGFR* wild type | Lind JS, Dingemans AM, Groen HJ, et al. A multicenter phase II study of erlotinib and sorafenib in chemotherapy-naive patients with advanced non-small cell lung cancer. Clin Cancer Res. 2010;16(11):3078-3087. | Martinelli E, Troiani T, Morgillo F, et al. Synergistic antitumor activity of sorafenib in combination with epidermal growth factor receptor inhibitors in colorectal and lung cancer cells. *Clin Cancer Res.* 2010;16(20):4990-5001. |
|  | Ganetespib-*ALK*+, crizotinib naïve | Socinski MA, Goldman J, El-Hariry I, et al. A multicenter phase II study of ganetespib monotherapy in patients with genotypically defined advanced non-small cell lung cancer. *Clin Cancer Res.* 2013;19(11):3068-3077. | Sang J, Acquaviva J, Friedland JC, et al. Targeted inhibition of the molecular chaperone Hsp90 overcomes ALK inhibitor resistance in non-small cell lung cancer. Cancer Discov. 2013;3(4):430-443. |
|  | Salirasib-*KRAS* mutant | Riely GJ, Johnson ML, Medina C, et al. A phase II trial of Salirasib in patients with lung adenocarcinomas with KRAS mutations. *J Thorac Oncol.* 2011;6(8):1435-1437. | Zundelevich A, Elad-Sfadia G, Haklai R, Kloog Y. Suppression of lung cancer tumor growth in a nude mouse model by the Ras inhibitor salirasib (farnesylthiosalicylic acid). *Mol Cancer Ther.* 2007;6(6):1765-1773. |
|  | Selumetinib-*KRAS* mutant | Janne PA, Shaw AT, Pereira JR, et al. Selumetinib plus docetaxel for KRAS-mutant advanced non-small-cell lung cancer: a randomised, multicentre, placebo-controlled, phase 2 study. *Lancet Oncol.* 2013;14(1):38-47. | Chen Z, Cheng K, Walton Z, et al. A murine lung cancer co-clinical trial identifies genetic modifiers of therapeutic response. *Nature.* 2012;483(7391):613-617. |
|  | Lapatinib-*EGFR* amplified | Ross HJ, Blumenschein GR, Jr., Aisner J, et al. Randomized phase II multicenter trial of two schedules of lapatinib as first- or second-line monotherapy in patients with advanced or metastatic non-small cell lung cancer. Clin Cancer Res. 2010;16(6):1938-1949 | Diaz R, Nguewa PA, Parrondo R, et al. Antitumor and antiangiogenic effect of the dual EGFR and HER-2 tyrosine kinase inhibitor lapatinib in a lung cancer model. *BMC cancer.* 2010;10:188. |
|  | Vandetanib-*RET-CCDC6* fusion | Yoh K, Seto T, Satouchi M, et al. Vandetanib in patients with previously treated RET-rearranged advanced non-small-cell lung cancer (LURET): an open-label, multicentre phase 2 trial. *The Lancet. Respiratory medicine.* 2017;5(1):42-50. | Suzuki M, Makinoshima H, Matsumoto S, et al. Identification of a lung adenocarcinoma cell line with CCDC6-RET fusion gene and the effect of RET inhibitors in vitro and in vivo. *Cancer Sci.* 2013;104(7):896-903. |
|  | Sunitinib-unselected patients | Heist RS, Wang X, Hodgson L, et al. CALGB 30704 (Alliance): A randomized phase II study to assess the efficacy of pemetrexed or sunitinib or pemetrexed plus sunitinib in the second-line treatment of advanced non-small-cell lung cancer. *J Thorac Oncol.* 2014;9(2):214-221. | Larrayoz M, Pio R, Pajares MJ, et al. Contrasting responses of non-small cell lung cancer to antiangiogenic therapies depend on histological subtype. *EMBO molecular medicine.* 2014;6(4):539-550. |
|  | Motesanib-unselected patients | Blumenschein GR, Jr., Kabbinavar F, Menon H, et al. A phase II, multicenter, open-label randomized study of motesanib or bevacizumab in combination with paclitaxel and carboplatin for advanced nonsquamous non-small-cell lung cancer. *Ann Oncol.* 2011;22(9):2057-2067. | Coxon A, Ziegler B, Kaufman S, et al. Antitumor activity of motesanib alone and in combination with cisplatin or docetaxel in multiple human non-small-cell lung cancer xenograft models. *Mol Cancer.* 2012;11:70. |
|  | Cediranib -unselected patients | Dy GK, Mandrekar SJ, Nelson GD, et al. A randomized phase II study of gemcitabine and carboplatin with or without cediranib as first-line therapy in advanced non-small-cell lung cancer: North Central Cancer Treatment Group Study N0528. *J Thorac Oncol.* 2013;8(1):79-88. | Wedge SR, Kendrew J, Hennequin LF, et al. AZD2171: a highly potent, orally bioavailable, vascular endothelial growth factor receptor-2 tyrosine kinase inhibitor for the treatment of cancer. *Cancer Res.* 2005;65(10):4389-4400. |
|  | Pazopanib-unselected patients | Altorki N, Lane ME, Bauer T, et al. Phase II proof-of-concept study of pazopanib monotherapy in treatment-naive patients with stage I/II resectable non-small-cell lung cancer. *J Clin Oncol.* 2010;28(19):3131-3137. | Tailor TD, Hanna G, Yarmolenko PS, et al. Effect of pazopanib on tumor microenvironment and liposome delivery. *Mol Cancer Ther.* 2010;9(6):1798-1808. |
|  | BI 2536-unselected patients | Sebastian M, Reck M, Waller CF, et al. The efficacy and safety of BI 2536, a novel Plk-1 inhibitor, in patients with stage IIIB/IV non-small cell lung cancer who had relapsed after, or failed, chemotherapy: results from an open-label, randomized phase II clinical trial. *J Thorac Oncol.* 2010;5(7):1060-1067. | Steegmaier M, Hoffmann M, Baum A, et al. BI 2536, a potent and selective inhibitor of polo-like kinase 1, inhibits tumor growth in vivo. *Current biology : CB.* 2007;17(4):316-322. |
|  | Axitinib-unselected patients | Schiller JH, Larson T, Ou SH, et al. Efficacy and safety of axitinib in patients with advanced non-small-cell lung cancer: results from a phase II study. *J Clin Oncol.* 2009;27(23):3836-3841. | Hu-Lowe DD, Zou HY, Grazzini ML, et al. Nonclinical antiangiogenesis and antitumor activities of axitinib (AG-013736), an oral, potent, and selective inhibitor of vascular endothelial growth factor receptor tyrosine kinases 1, 2, 3. *Clin Cancer Res.* 2008;14(22):7272-7283. |
|  | Trametinib-*KRAS* mutant | Blumenschein GR, Jr., Smit EF, Planchard D, et al. A randomized phase II study of the MEK1/MEK2 inhibitor trametinib (GSK1120212) compared with docetaxel in KRAS-mutant advanced non-small-cell lung cancer (NSCLC)dagger. *Ann Oncol.* 2015;26(5):894-901. | Tao Z, Le Blanc JM, Wang C, et al. Coadministration of Trametinib and Palbociclib Radiosensitizes KRAS-Mutant Non-Small Cell Lung Cancers In Vitro and In Vivo. *Clin Cancer Res.* 2016;22(1):122-133. |

**Supplemental Table 2:** Excluded approved drug- indication pairs and excluded unapproved drugs

| **Excluded Approved Drug-Indication Pair** | **Excluded Unapproved Drug** |
| --- | --- |
| Afatinib-*EGFR* exon 21 L858R mutation  Crizotinib-*ROS1* positive  Erlotinib-*EGFR* exon 19 deletion  Erlotinib- *EGFR* exon 21 L858R mutation  Gefitinib- *EGFR* exon 21 L858R mutation  Trametinib + Dabrafenib- *BRAF* V600E mutation | Buparlisib  Cabozantinib  Dasatinib  Dinaciclib  Fostamatinib  Linifanib  Icotinib  Neratinib  XL-647 |

**Supplemental Table 3:** For 10 randomly selected drug-indication pairs, there is no significant difference between data utilized in the study cohort and the data collected by a reviewer who attempted to replicate the results. The process of replicating the data required identifying 18 publications (one preclinical study and one clinical trial produced data for 2 drug-indication pairs). The reviewer identified 17/18 publications of the publications used in the study. For the sole discrepancy, the reviewer’s publication would have been correct due to the presence of an additional 3 citations on Google Scholar. Therefore, we included the reviewer’s discrepant data to see if a minor discrepancy would create a statistically significant difference in the results. However, there remained no significant difference between mean study and mean replication data.

| **Clinical Characteristic** | **Study Cohort (10 randomly selected DIPs)**  **Mean (standard error)** | **Independent Reviewer (10 randomly selected DIPs)**  **Mean (standard error)** | **P value** |
| --- | --- | --- | --- |
| N-intention to treat | 143.7 (26.33) | 143.9 (26.37) | 0.9958 |
| N-selected patient population | 64 (20.66) | 65.4 (20.94) | 0.9626 |
| % Stable Disease | 32.36 (4.527) | 35.01 (4.391) | 0.6860 |
| % Female | 59.15 (3.201) | 59.15 (3.201) | >0.9999 |
| Median age | 60.6 (1.352) | 60.6 (1.352) | >0.9999 |
| % White | 63.09 (7.74) | 63.09 (7.74) | >0.9999 |
| % Asian | 39 (11.24) | 39 (11.24) | >0.9999 |
| % ECOG = 0 | 41.76 (4.337) | 41.76 (4.337) | >0.9999 |
| % ECOG = 1 | 53.96 (4.481) | 53.96 (4.481) | >0.9999 |
| % ECOG = 2 | 3.714 (1.835) | 3.714 (1.835) | >0.9999 |
| % Patients who are current or former smokers | 40.29 (8.903) | 40.29 (8.903) | >0.9999 |
| % Adenocarcinoma | 87.43 (4.49) | 87.88 (4.356) | 0.9442 |
| % Squamous carcinoma | 5.871 (1.958) | 5.871 (1.958) | >0.9999 |
| % Patients with CNS metastasis | 50.5 (5.485) | 50.5 (5.485) | >0.9999 |
| % Patients who received prior chemotherapy | 66.38 (14.99) | 59.4 (13.57) | 0.7353 |
| % Stage IV | 87.4 (4.354) | 87.4 (4.354) | >0.9999 |
| ORR | 46.97 (8.921) | 46.97 (8.921) | >0.9999 |
| **Pre-clinical metric** | **Study Cohort (10 randomly selected DIPs)**  **Mean (standard error)** | **Independent Reviewer (10 randomly selected DIPs)**  **Mean (standard error)** | **P value** |
| TC Ratio | 13.75 (5.656) | 14.1 (6.644) | 0.9684 |
| Duration animal monitoring (days) | 57.4 (22.75) | 56.5 (22.92) | 0.9781 |
| N mice | 7.3 (0.6333) | 7.5 (0.6191) | 0.8239 |
| N pubs before date of Phase II | 121.8 (29.52) | 119.6 (29.79) | 0.9587 |
| Mouse model* | HC = 90%  PDX = 0%  GEM = 0%  MC = 10% | HC = 100%  PDX = 0%  GEM = 0%  MC = 0% | * |
| Drug is approved for other cancers before Phase II? | 30% | 30% | >0.9999 |

* Chi square test was not performed due to the presence of zero as an expected value. All mouse models matched except for 1/10. The sole discrepancy resulted from the selection of a different publication by the reviewer than was utilized in the study. HC = human cell line xenograft, PDX = patient derived xenograft, GEM = genetically engineered mouse model, MC = mouse cell line xenograft

**Supplemental Table 4:** There is minimal effect of mouse-patient matching type on the relationship between preclinical metrics and ORR. P values in bold are significant. When Unselected to Unselected drugs (n = 6, “Matched Drugs”) were removed from the analysis, similar differences in preclinical metrics between ORR High and ORR Low drugs remained.

| Variable | All drugs- ORR ≥ 50 v. ORR < 50 | Matched drugs- ORR ≥ 50 v. ORR < 50 |
| --- | --- | --- |
|  |  |  |
| T/C Ratio | **0.0043** | **0.0110** |
| T/C Ratio = 0 | **0.0143** | 0.1063 |
| Mouse model | 0.2483 | 0.5357 |
| Duration animal monitoring (days) | 0.8259 | 0.7009 |
| N mice | **0.0310** | **0.0499** |
| N pubs before date of Phase II | 0.1267 | 0.2606 |
| Drug is approved for other cancers before Phase II | 0.0821 | 0.1436 |
| Matching type | **0.0065** | 0.3494 |

**Supplemental Table 5:** There is minimal effect of mouse-patient matching type on the relationship between preclinical metrics and ORR. P values in bold are significant. When Unselected to Unselected drugs (n = 6, “Matched Drugs”) were removed from the analysis, similar differences in preclinical metrics between Approved and Unapproved drugs remained.

| Variable | All Drugs-Approved vs. Unapproved | Matched Drugs-Approved vs. Unapproved |
| --- | --- | --- |
|  |  |  |
| TC Ratio | **0.0303** | 0.0914 |
| TC Ratio = 0 | 0.1837 | 0.6479 |
| Mouse model | 0.5700 | 0.8868 |
| Duration animal monitoring (days) | 0.6073 | 0.9843 |
| N mice | **0.0079** | **0.0125** |
| N pubs before date of Phase II | **0.0439** | 0.1013 |
| Drug is approved for other cancers before Phase II | **0.0176** | **0.0205** |
| Matching type | **0.0066** | 0.0777 |

**Supplemental Table 6:** By multivariate logistic regression analysis, no pre-clinical metrics are independently associated with approval status.

| **Approved Categorical All Drugs-Probability modeled is “Drug is unapproved”**  Testing H_0_ Beta = 0: Chi square = 35.9935 , p <.0001 | | |
| --- | --- | --- |
| **Predictor Variable** | **β Estimate (standard error)** | **p value** |
| TC Ratio | 0.289 (2.594) | 0.9112 |
| N mice | 4.812 (19.854) | 0.8085 |
| N pubs before date of Phase II | -0.124 (0.381) | 0.7448 |
| Drug is approved for other cancers before Phase II? | 31.282 (194.4) | 0.8721 |
| Matching Type | 2.768 (16.822) | 0.8693 |
|  |  |  |
| **Approved Categorical Matched Drugs-Probability modeled is “Drug is unapproved”**  Testing H_0_ Beta = 0: Chi square = 30.7370 , p = <.0001 | | |
| **Predictor Variable** | **β Estimate (standard error)** | **p value** |
| TC Ratio | 0.171 (0.878) | 0.8452 |
| N mice | 4.223 (5.478) | 0.4408 |
| N pubs before date of Phase II | -0.082 (0.113) | 0.4695 |
| Drug is approved for other cancers before Phase II? | 11.827 (41.967) | 0.7781 |
| Matching Type | -9.372 (42.096) | 0.8238 |
|  |  |  |

**Supplemental Table 7:** By multivariate logistic regression analysis, no pre-clinical metrics are independently associated with achieving an ORR threshold value of 50%.

| **ORR Categorical All Drugs-Probability modeled is ORR <50%**  Testing H_0_ Beta = 0: Chi square = 27.6719, p < 0.0001 | | |
| --- | --- | --- |
| **Predictor Variable** | **β Estimate (standard error)** | **p value** |
| TC Ratio | 0.132 (0.107) | 0.2152 |
| N mice | 1.291 (0.913) | 0.1575 |
| N pubs before date of Phase II | 0.001 (0.009) | 0.9403 |
| Drug is approved for other cancers before Phase II? | 2.347 (2.134) | 0.2713 |
| Matching Type | 4.877 (2.908) | 0.0935 |
|  |  |  |
| **ORR Categorical Matched Drugs-Probability modeled is ORR <50%**  Testing H_0_ Beta = 0: Chi square = 16.0726, p = 0.0066 | | |
| **Predictor Variable** | **β Estimate (standard error)** | **p value** |
| TC Ratio | 0.131 (0.106) | 0.2165 |
| N mice | 1.268 (0.915) | 0.1655 |
| N pubs before date of Phase II | 0.001 (0.009) | 0.9342 |
| Drug is approved for other cancers before Phase II? | 2.301 (2.148) | 0.2842 |
| Matching Type | 4.759 (3.009) | 0.1137 |
|  |  |  |

**Supplemental Figure 1:** Patient characteristics correlate with ORR in phase II clinical trials. There is a significant positive correlation between ORR and the percent of patients in the phase II study with female sex **(1A),** Asian ethnicity **(1B)**, and adenocarcinoma **(1C)**. There is a significant negative correlation between ORR and the percent of patients in the phase II study with white ethnicity **(1D)**, squamous histology **(1E)**, and a history of smoking **(1F)**.
